# Novel Insights into Adipogenesis from Omics Data

**DOI:** 10.2174/092986709788803132

**Published:** 2009-08

**Authors:** Andreas Prokesch, Hubert Hackl, Robab Hakim-Weber, Stefan R Bornstein, Zlatko Trajanoski

**Affiliations:** 1 Institute for Genomics and Bioinformatics, Graz University of Technology, Graz, Austria; 2 Department of Internal Medicine, Technical University Dresden, Dresden, Germany

**Keywords:** Adipogenesis, obesity, gene-expression profiling, proteomics, genome-wide location analysis, data integration.

## Abstract

Obesity, the excess accumulation of adipose tissue, is one of the most pressing health problems in both the Western world and in developing countries. Adipose tissue growth results from two processes: the increase in number of adipocytes (hyperplasia) that develop from precursor cells, and the growth of individual fat cells (hypertrophy) due to incorporation of triglycerides. Adipogenesis, the process of fat cell development, has been extensively studied using various cell and animal models. While these studies pointed out a number of key factors involved in adipogenesis, the list of molecular components is far from complete.

The advance of high-throughput technologies has sparked many experimental studies aimed at the identification of novel molecular components regulating adipogenesis. This paper examines the results of recent studies on adipogenesis using high-throughput technologies. Specifically, it provides an overview of studies employing microarrays for gene expression profiling and studies using gel based and non-gel based proteomics as well as a chromatin immunoprecipitation followed by microarray analysis (ChIP-chip) or sequencing (ChIP-seq). Due to the maturity of the technology, the bulk of the available data was generated using microarrays. Therefore these data sets were not only reviewed but also underwent meta analysis.

The review also shows that large-scale omics technologies in conjunction with sophisticated bioinformatics analyses can provide not only a list of novel players, but also a global view on biological processes and molecular networks. Finally, developing technologies and computational challenges associated with the data analyses are highlighted, and an outlook on the questions not previously addressed is provided.

## INTRODUCTION

Since the cloning of the obese gene in 1994 [1] and the subsequent functional characterization of its product, leptin, it has become increasingly evident that adipose tissue is a key organ in the regulation of the body’s energy homeostasis rather than a passive storage of fat. Adipocytes in white adipose tissue (WAT) have been shown to secrete cytokines and, as more recently published, lipid signals such as C16:1n7-palmitoleate in response to the nutritional status of the organism [2]. These adipocytokines and lipokines communicate with other metabolically active tissues, such as liver, muscle, and the gut, to equilibrate metabolites throughout the body [3]. Perturbations of these communications can lead to disturbances in the regulation of whole body energy homeostasis. In particular, increased WAT mass (especially in visceral depots) is associated with insulin-resistance, which is a major cause of diabetes, hypertension and cardiovascular disease [4,5]. These obesity-related disorders are major health and economic concerns for the modern society, including not only the western societies but also rapidly developing countries such as China [6] and India [7]. The development of WAT is the result of two processes: the increase in number of adipocytes (hyperplasia) that develop from precursor cells, and the growth of individual fat cells (hypertrophy) due to incorporation of triglycerides. As recently shown by Spalding *et al.* total body fat mass in humans is determined by adipocyte number and size [8]. Interestingly, it could be shown that the total number of adipocytes increases only in childhood and adolescence, while staying constant in adulthood [8]. By measuring ^14^C incorporated in DNA during cold war atomic bomb testing, this study estimated the annual turnover of fat (replacement of lost cells with new adipocytes derived from precursor cells) in adults to be ~10% [8]. These results, along with others, emphasize the importance of the process of adipogenesis and circumstantiate the need for comprehensive understanding of this process at a molecular level.

**Fig. (1):**
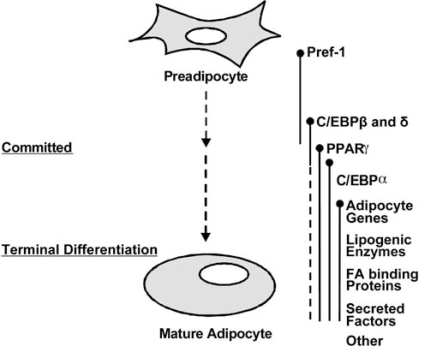
Development of mature adipocytes from preadipocytes. (Adopted from [119]). Dlk1 (Pref-1), Cebpb and Cebpd are expressed at the early stages of the differentiation process. After commitment, Pparg and Cebpa are upregulated and target many genes relevant for the function of mature adipocytes.

The development of fat cells is a process that can be modeled in cell culture. The mouse pre-adipocyte cell line 3T3-L1 is a readily-available, well-described model for the adipocyte differentiation process (Fig. **1**). Upon defined hormonal induction 3T3-L1 cells can be induced to undergo adipogenesis to a point where nearly all cells are filled with lipid droplets and can respond to physiological signals (e.g. glucose uptake upon insulin treatment or cAMP activation and lipolysis *via *β-adrenergic stimuli). Other mouse cell lines are also suitable for modeling adipogenesis *in vitro* (e.g. 3T3-F442A, NIH-3T3 and OP9 [9]) as are primary cells derived from mouse embryo fibroblasts (MEFs) or mesenchymal stem cells (for example, isolated from bone marrow stroma). Human cell culture models are primarily obtained from bone-marrow biopsies or from adipose tissue liposuctions [10]. Both human [11] and mouse [12] embryonic stem cells can also be coaxed into an adipogenic differentiation pathway. These *in vitro* models have been utilized for more than 40 years to discover the molecular players involved in adipogenesis. Countless biochemical studies have been performed to define the transcriptional events governed by the two master regulators of adipogenesis [13]: Peroxisome proliferators activated receptor gamma (Pparg) and CCAAT/ enhancer-binding protein alpha (Cebpa).

Encouraged by the appearance of high-throughput technologies (initially microarrays that are now utilized as standard lab tools) and by the availability of sequencing data on many species, omics technologies sparked the interest to perform a system-wide analysis on the biological system of interest. Multiple variables can be measured in parallel and on different molecular levels by using technologies such as transcriptomics (mRNA levels), genome-wide location analysis (DNA-protein interactions), proteomics (protein expression levels), epigenomics (e.g. histone modifications) and metabolomics (small molecules as intermediary metabolites).

This paper examines the results of large-scale studies on adipogenesis using high-throughput technologies. Specifically, it provides an overview of studies employing microarrays for gene expression profiling and studies using gel based and non-gel based proteomics as well as a chromatin immunoprecipitation followed by microarray analysis (ChIP-chip) or sequencing (ChIP-seq) for the identification of target genes of transcription factors. Due to the maturity of the technology, the bulk of the available data was generated using microarrays. Therefore these data sets were not only reviewed but underwent meta analysis.

## GENE EXPRESSION PROFILING OF ADIPOGENESIS

Large- scale gene expression profiling is a discovery-driven approach used to identify candidate genes, which are then subjected to further in-depth functional studies. Moreover, this technology can be utilized to characterize molecular effects in silencing, knock-out or over- expression strategies of these candidate genes in cell models, tissues or organisms. A number of expression profiling studies [14-36] using microarrays were performed to monitor the global gene expression profiles during *in vitro* adipocyte differentiation in different cell models and organisms as summarized in Table **1**. These studies used the most prominent model (the 3T3-L1 cell line) and three different array technologies: spotted arrays, commercial oligo-nucleotide microarrays, and spotted cDNA arrays. Guo and Liao [14] used a spotted array filter-based system to compare gene expression levels in differentiated 3T3-L1 cells (induced by a standard hormone cocktail used in most studies including dexamethasone, isobutylmethylxanthine, and insulin (DMI) in presence of calf bovine serum) to those of 3T3-L1 preadipocytes. Commercial oligo-nucleotide microarrays (Affymetrix GeneChips) were often employed to study gene expression profiling of adipogenesis and the studies differ mostly in experimental design and selection of time points. While some of those studies focus on molecular events at early stages, others cover the whole adipocyte differentiation process including preconfluent stage, growth arrest, mitotic clonal expansion, and terminal differentiation (see Table **1**). Moreover, the effects of different components of the differentiation cocktail on gene expression were addressed, as was the question of which genes are affected by adding Pparg activators, like Rosiglitazone, to identify potential target genes for Pparg. cDNA microarrays were used to study the whole differentiation process not only to discover novel molecular players but also to obtain a global view on biological processes and molecular networks during adipogenesis [22]. While the study in mice used primary and immortalized embryonic fibroblasts and derived cell lines like the 3T3-L1 and 3T3-F442A, the studies in humans focused on primary preadipocyte and adipocyte cells, mesenchymal stem cells from bone marrow and adipose tissue. Most mouse cells undergo one or two rounds of clonal expansion during adipocyte differentiation - an event that could not be observed in human adipocyte differentiation. Those differences were mirrored in the expression profiles of genes known to be involved in the cell cycle (e.g. cyclin B1), with a sharp increase in the 3T3-L1 cell line at 24h [22], in MEFs at 24h and at 72h after hormonal induction (unpublished observations) and only marginal changes in gene expression during hMADs adipocyte differentiation [35].

An important aspect of microarray analyses is the quality of data. Additional systematic biases or effects can be introduced for integrating gene expression data if the studies were performed in different laboratories using different platforms or even different species. An exhaustive analysis and comparison of commonly used microarray platforms by a multicenter consortium (MAQC) showed - contrary to earlier reports [37,38] - acceptable concordance between the platforms [39]; however, there is a necessity for careful control of biological samples and close adherence to standard protocols [40]. There is also an imminent problem with using varying platforms: namely the different probes, probe sequences and annotations. More confidence in analyzed gene expression levels can be gained if the levels are confirmed using different low-scale or medium-scale technologies, like quantitative real-time reverse transcriptase polymerase chain reaction (qPCR), as was done for many adipogenesis studies. For the publication of studies based on microarray data, a prerequisite of most journals is the submission of the data and experimental parameters to a public repository. This affords the scientific community the opportunity to extract genes potentially involved in adipocyte differentiation from: (1) expression data reported explicitly in publications or supplemental data; (2) pre-processed or raw data in public repositories or gene expression databases after adequate analysis (normalization); or (3) integrated expression profiles over different experiments by ‘meta-analyses’ similar to the meta-analyses in clinical research, in which a generalized hypothesis in a systematic review is deduced from the analyses of multiple studies. The results of this type of meta-analysis performed for the purpose of this paper are given in Fig. (**1**). In addition to the key transcription factors like Cebpa and Pparg, enzymes and other uncharacterized genes were identified. A compendium from some of the relevant datasets - discussed above - are provided by the Genomics Of Lipid-associated Disorder database (GOLD.db) [41].

Many genes identified by expression profiling using microarrays and different cell models are potentially involved in the regulation of fat cell development. To discuss all candidates is beyond the scope of this article; it instead focuses on a handful of candidates, selected for their gene expression profiles and further studied in detail to gain novel insights into the molecular mechanisms of the fat cell development process (see also other reviews [13,42,43]). Since the adipocyte differentiation is driven by a transcriptional cascade, a major goal is the identification of transcriptional regulators involved or even required in the (early) adipogenesis process, moderated by molecular events previous to the activation or direct regulation of the key regulators Pparg and Cebpa, (e.g. by binding to its corresponding promoter sequences). One candidate selected from results based on a microarray study in 3T3-L1 [15,44] is Klf4, which was shown to function as an immediate early regulator of adipogenesis by inducing Cebpb and is required for adipogenesis [44]. Several members of the Krüppel- like factor family have previously been implicated in adipogenesis: Klf6 [45] and Klf15 [46] have both been shown to promote adipogenesis and Klf5 [47] is necessary for adipocyte differentiation and acts by transactivating Pparg. Targeted disruption of the Klf3 gene reveals a role in adipogenesis and Klf2 inhibits Pparg expression and adipogenesis [48]. Expression profiles of Klf9 are modulated during 3T3-L1 adipocyte differentiation (see e.g. [22]); however, a regulatory function has yet to be confirmed. Induction of Klf9 in NIH3T3 cells could not induce differentiation into adipocytes [46]. All of these factors are thought to function by recruiting different coactivators or repressors [13]. Expression levels from different reanalyzed experiments for the Klfs and other candidates (described below) are summarized as heat maps in Fig. (**1**). Another candidate from these microarray results is the zinc finger-containing transcription factor Egr2 (Krox20). The expression of Egr2 is activated very early after induction and stimulates adipogenesis at least in part through activating Cebpb by binding to its promoter [49]. The (orphan) nuclear receptor Nr4a1 (Nur77) is also known as an immediate early gene as indicated in the expression profiles of several adipogenesis studies. Overexpression of Nr4a1 blocks adipogenesis in 3T3-L1 cells ([50] and unpublished observation from this lab), whereas Nr4a1 might not be required for adipogenesis according to contradictory results with Nr4a1 knock down by siRNA in 3T3-L1 cells [51,52]. Nr4a1 could be involved in the mitotic clonal expansion [52] and the other NR4A family members also have pleiotropic physiological roles including energy metabolism such as regulation of lipolysis in skeletal muscle cells [53] and hepatic gluconeogenesis [54]. Another nuclear hormone receptor involved in adipogenesis is Nr1h3 (LXRalpha). A broader role of Nr1h3 in regulation of metabolism in adipocytes was suggested and the effects of Wnt–signaling in adipocyte differentiation were studied in a timed series microarray experiments of 3T3-L1 cells and retroviral infected 3T3-L1 cells encoding Wnt1 [16]. It is known that liver X receptors (LXRs) regulate cholesterol and fatty acid metabolism in liver tissue and macrophages. Recently it was also shown that activated Nr1h3 stimulate adipocyte differentiation through induction of Pparg expression but it is not required for adipocyte differentiation [55]. A nuclear receptor gene expression atlas during the differentiation of 3T3-L1 cells, assessed using qPCR, also showed the importance of other nuclear receptors such as the Nr2f2 (COUP-TF2) in adipogenesis [35,56,57]. The role of Ebf1 (O/E-1), a helix-loop-helix transcription factor, was studied in adipocytes with microarray analysis of Ebf1 over-expression in NIH-3T3 cells [26]. Further experiments helped place Ebf1 within the known transcriptional cascade of adipogenesis [58]. By the year 2000, it was shown that Gata2 and Gata3 are specifically expressed in adipocyte precursors and their down-regulation sets the stage for terminal differentiation [59]. This type of expression profile could be confirmed later on with microarray experiments. A role for transcriptional coregulators in the control of energy homeostasis could be shown by knock-out of the co-repressor Nrip1 (RIP140) in adipocytes [28].

Microarray analyses reveal not only transcription factors but also enzymes as important regulators for adipogenesis. Xanthine dehydrogenase (Xdh, XOR) could be identified as a novel regulator of adipogenesis and Pparg activity and as essential for the regulation of fat accretion [60]. In this analysis, emphasis was given to genes whose expression was limited to the first 24 hours after initiation of differentiation and candidate genes were ranked based on an algorithm modeling the complexity of each gene-expression profile [60]. Loss of function of Stearoyl-CoA desaturase (Scd1) - a central lipogenic enzyme catalyzing the synthesis of monounsaturated fatty acids - protects mice against adiposity. While another family member with the similar amino acid sequence Scd2 is required for Pparg expression and adipogenesis in cultured 3T3-L1 cells, Scd1 is not. Enzymes for fatty acid desaturation as well as factors for fatty acid elongation are differentially expressed during adipocyte differentiation like Elovl6 [22]. Recently, the enzyme adipose triglyceride lipase Pnpla2 (ATGL), which catalyzes the initial step in triglyceride hydrolysis, was discovered [61] and confirmed by microarrays [22]. A microarray study on the differential transcriptional modulation of biological processes in Pnpla2 deficient mice was subsequently published [62].

In summary, the advantages of using a microarray screening process to gain novel mechanistic insights in adipogenesis are three-fold. First, as described above, novel characterized candidate genes could be identified based on their expression profiles and confirmed by further functional studies. Second, also not characterized genes with modulated expression profile can be detected. The RIKEN mouse gene encyclopedia project is a systematic approach to determine the full coding potential of the mouse genome and involves collection and sequencing of full-length complementary DNAs and physical mapping of the corresponding genes to the mouse genome [63]. The annotation of many genes is based on this RIKEN approach (see Refseq [64] or FANTOM [65]) and cDNA arrays in particular (typical EST length ~1-1.5 kbp) provide the opportunity to study transcripts with high sequence similarity to those RIKEN genes and to elucidate new genes involved in the molecular mechanisms of adipocyte differentiation. Finally, large-scale gene expression profiling including the study of many transcripts makes it possible to obtain a global view on biological processes and molecular networks during adipogenesis.

## PROTEOMICS

The maturity of the microarray technology and the focus on the delineation of the transcriptional program of adipogenesis resulted in >20 studies conducted using this approach. In contrast, there are only a handful of published studies using proteomic approaches for identifying proteins during the differentiation of 3T3-L1 adipocytes. This is partly due to the complexity of the proteome with estimated >1.000.000 individual species and partly due to the limitations of the available technologies. Neither gel-based nor non gel-based techniques can currently detect molecules at the required sensitivity range of several orders of magnitude. Hence, the published studies report only a fraction of the adipocyte proteome and secretome (entire complement of secreted proteins).

Protein profiling during adipogenesis was performed with gel-based approaches using two-dimensional gel electrophoreses for separation and subsequent MALDI-TOF/MS (Matrix-assisted laser desorption/ionization – time of flight/mass spectrometry) for protein identification [66-70] as well as a non gel-based method using LC-MS/MS (liquid chromatography coupled with tandem mass spectrometry) [71]. The gel-based studies used either mouse 3T3-L1 models [67,68,70] or human mesenchymal stem cells [66,69] and reported between 8 and 2000 protein species whereas a non gel-based study [71] identified approximately 3300 proteins. The analysis of the depth and coverage of the 3T3-L1 adipocyte proteome compared to the liver organelle proteome map and to six mouse tissues showed that two-thirds of the proteins overlapped [71]. These proteins are candidates for the “core proteome” whereas around 1000 proteins were adipocyte specific. By comparing microarray data with the proteomic data, about 28% (2182 microarray probes out of the 7656) could be mapped to the identified proteome. However, further conformation in tissues samples as well as characterization of the candidates in a functional assay has not yet been performed.

It has become evident in recent years that fat tissue is an organ secreting large number of molecules including signaling metabolites, chemokines, and hormones. Two recent studies [72,73] addressed this issue and profiled the adipocyte secretome. In addition to a number of previously reported secreted factors like adipsin or adipocyte complement-related protein 30 kDa, four novel molecules were identified in the study by Kratchmarova *et al*: Pigment epithelium-derived factor (Serpinf1) secreted in preadipocytes, hippocampal cholinergic neurostimulating peptide (Pebp1), neutrophil gelatinase-associated lipocalin (Lcn2), and haptoglobin (Hp) in mature adipocytes. In another gel-based study using human cells, 170 individual proteins were detected [73]. Comparison of these data with reported secretomes showed varying similarities ranging between 4% (3T3-L1 secretome) and 49% (human lysates), reflecting the methodological and technical differences in proteomic studies.

## CHROMATIN IMMUNOPRECIPITATION (CHIP) TO DISCOVER TARGETS OF ADIPOGENESIS-RELEVANT TRANSCRIPTION FACTORS

Chromatin immunoprecipitation (ChIP) is a method for assessing direct DNA-protein interaction between transcription factors and their respective binding sites [74]. Immunoprecipitation with an antibody directed against the transcription factor of interest is performed on a nuclear extract of cross-linked chromatin. The read-out of such an experiment can be generated using gel electrophoresis after PCR amplification or with qPCR using primers specific to promoter regions of putative or known target genes. This approach was taken in many studies for detecting binding of adipogenic transcription factors to promoters of single genes. For example, in 3T3-L1 cells during adipogenesis, physical binding was shown for Pparg in the Cat [75], Lipe [76], and G0s2 [77] promoter and for Cebpa in the Cd36 [78] promoter, in the Dgat2 [79] promoter and in promoters of several adipokines (Resistin, adiponectin and leptin) [80]. Alternatively, hybridization of labeled immunoprecipitated samples to a microarray containing probes that represent a selection of regulatory segments or that are tiling the whole genome makes this method amenable to high-throughput analysis. The latter method is known as ChIP-chip [81,82]. More recently, next-generation sequencing technology was applied to sequence the DNA fragments obtained from a ChIP experiment (ChIP-seq). Despite the advantage of obtaining direct binding information from ChIP studies it cannot automatically be inferred that this binding is functional, i.e. it yields to expression of the target genes. This can be partly explained by the fact that transcription of many genes depends on the synergistic action of several transcription factors. It is therefore essential to combine such binding data with transcriptomics data to ensure that binding to a target region also leads to an effect on the mRNA level [83].

Up to date five studies have been published that employ ChIP-chip [84,85], ChIP-seq [86,87], or both technologies [88] during adipogenesis, all performed with 3T3-L1 cells. One report, by Nakachi *et al*. [84], integrates gene expression data with ChIP-chip data (obtained from promoter chips) and computational binding site predictions to identify Pparg target genes during adipogenesis in 3T3-L1 cells. Using an antibody that recognizes both Pparg isoforms (Pparg1 and Pparg2) they could report 167 Pparg-bound genes, including 20 genes that are bound by Pparg and are upregulated during adipogenesis. Five of those 20 genes - two *bona fide* target genes (Cfd, Fabp4) and three novel target genes (Tmem143, Hp, 1100001G20Rik) - were confirmed as activated by Pparg by means of luciferase assays in NIH 3T3 cells [84].

A more comprehensive and unbiased approach is the use of whole-genome tiling arrays as reported by Lefterova *et al*. [85]. Mature adipocytes (d10) were subjected to ChIP using antibodies against Pparg, Rxra, Cebpa and Cebpb. Several surprising outcomes were revealed in this study. First, 5299 identified Pparg-bound regions were located mainly in distal intergenic regions (more than 1kbp away from 5’ or 3’ ends of genes; 52%) and in introns (32%). This might explain the relatively low number of identified Pparg-bound promoters in the Nakachi study where microarrays with probes only covering the proximal promoter regions were used. Second, Rxra (the major heterodimerization partner of Pparg) binding sites were found in proximity to a randomly selected set of Pparg binding sites in 98% of the observed cases. Third, bioinformatics analyses predicted a high degree of potential C/EBP binding sites in the vicinity of Pparg binding sites. Subsequent ChIP-chip experiments with a Cebpa antibody identified 16,760 Cebpa binding sites with a genomic location distribution similar to Pparg. Sixty-three percent of the Pparg binding sites (3,350) overlap with Cebpa binding (defined by at least 1 bp overlap between the 1,000bp-long bound regions). Fourth, more than 60% of upregulated genes (from gene expression data) contained binding sites of both Pparg and Cebpa within 50kb of their upstream regions. And fifth, ChIP-chip for Cebpa and Cebpb using a custom array containing Pparg binding regions showed nearly identical binding profiles (99.1%), pointing to functional promoter occupancy of Cebpb in late adipogenesis and its redundancy with Cebpa. This was further strengthened by the fact that only silencing of both factors lead to a decrease in expression of some target genes. However, when Pparg is silenced in addition the decrease in target gene expression is even more pronounced, thus presenting strong evidence for the synergistic adipogenic action of these three factors.

Simultaneously, a ChIP-seq study on Pparg and Rxra binding during 3T3-L1 adipogenesis was published [86]. At day six of adipogenesis 5236 Pparg:Rxr heterodimer binding sites were reported. This number, as well as the genomic distribution pattern of the binding sites (>50% found in intergenic regions), is in good concordance to the study from Lefterova *et al. *[85]. However, an additional value is the assessment of Pparg and Rxr binding at several time points during adipogenesis (d0, d1, d2, d3, d4, d6). This experimental design revealed that heterodimer composition changes during the course of adipogenesis with many Rxr binding sites in early time points having no respective Pparg binding sites. This suggests that in early adipogenesis Rxr has other binding partners as was shown for Ppard [86]. Another interesting outcome of this time series experiment is that >94% of Pparg binding sites at day zero to day four are to be detected at day six. Finally, determining genome-wide RNA polymerase II occupancy as a measure for transcriptional activity showed that Pparg:Rxr binding sites are particularly enriched in the vicinity of upregulated genes.

Another study was employing ChIP followed by pair end-tagging (PET) sequencing technology to identify 7821 Pparg and Rxr binding sites [87]. Combining this binding data with gene expression (expression profiles of differentiated and Pparg-silenced 3T3-L1 cells) and validation studies (ChIP-qPCR) yielded 75 high-confidence Pparg:Rxr target genes. Four out of six tested binding elements showed substantially increased luciferase activity when cloned in front of a reporter construct, indicating that they are potent transcriptional activation elements. Further, in an siRNA screen designed to knock-down 20 putative Pparg:Rxr target genes, six could be shown to impair lipid accumulation when silenced. For a list of validated Pparg:Rxr targets emerging from this study see Table **2**.

Finally, Wakabayashi *et al*. [88] arrived at a similar global view of Pparg:Rxra binding in 3T3-L1 cells like the afore-mentioned genome-wide location studies. To take their work one step further, however, they focused on a group of SET domain proteins that were identified by their ChIP-chip experiments. These proteins are known to catalyze methylation of histones on lysin residues. In particular, generating an H4K20me1 modification map across the genome (using ChIP-seq) they could show that Setd8 regulates the expression of Pparg and some of its target genes through H4K20 mono-methylation. Thus, in this study a link between transcriptional regulation and epigenetic modulation, as well as the positive feedback loop between these processes, is presented.

One pressing question emerging from these and other genome-wide location studies is, if and how binding sites that are far away from 5’ ends of genes (up to several 10kb) can confer transcriptional activation. In an elegant study Tomura *et al*. used the example of the Resistin promoter and showed functional relevance of a region that is ~8.8kb upstream of the transcription start site and contains three Cebp and one Pparg binding sites [89]. Still it is not clear by which mechanism this long-range interactions between distant binding sites and the transcription start sites can occur in living cells.

These genome-wide location studies are of great interest and provide a high-confidence part list of the adipogenic process. Unfortunately, a direct comparison of these studies is not possible due to the different protocols, antibodies, platforms, and technologies used and because of non-standardized bioinformatics analyses.

## MEDIUM-SCALE METHODS APPLIED TO ADIPOGENESIS

In addition to the described omics technologies, three other medium-scale methods were employed to characterize the process of fat cell development and to identify new candidates in this process: RNAi-screens, DNase I hypersensitivity (Table **2**), and a chemical biology approach.

In one report, short interfering RNA-based screens were conducted in 3T3-L1 cells using insulin-stimulated glucose uptake (a functional characteristic of mature adipocytes) as a read-out. By minituarizing the procedure it was possible to perform 30-50 gene silencings per week [90]. As reported in a series of publications this approach lead to a characterization of required proteins (summarized in [91]) and to a determination of essential kinases [92] in the insulin pathway of mature adipocytes. Further, this RNA interference screen yielded a number of novel players during adipogenesis, such as Cidec [93], Scd2 [94] and Nrip1 [95]. In the studies on Scd2 and Nrip1 Affymetrix GeneChip analysis was performed on differentiated 3T3-L1 cells transfected with either scrambled or siRNA constructs to elucidate the pathways affected by the silencing of these genes and to place them in the transcriptional cascade.

Another “top-down” approach for identifying as yet unknown players in adipogenesis was the use of high-throughput DNase I hypersensitivity analysis in conjunction with a computational strategy to identify differentiation-dependent *cis*- and *trans*-acting factors. The principle of DNase I hypersensitivity assays is that regulatory genomic elements (e.g. promoters, enhancers) are more accessible to digestion by nucleases than to sites of inactive chromatin [96]. The digested DNA can then be subjected to measurement using southern blotting or qPCR. In their studies, Rosen and colleagues used 268 pre-selected primer pairs mapped to upstream regions of 27 adipogenesis-relevant genes and performed qPCR on DNase I digested nuclei derived from 3T3-L1 cells prior to and seven days after differentiation induction. The identified DNase hypersensitive sites were then computationally analyzed to yield overrepresented sequence motifs [97]. One of the highest scoring motifs was a binding site for the interferon regulatory factor (Irf) family. Consequently, all nine known Irfs were tested for their expression in adipose tissue and during 3T3-L1 differentiation, and for their binding to the predicted *cis* elements. Finally, some Irfs were shown to be potent inhibitors of adipogenesis [97]. Another candidate that emerged from this approach was the nuclear receptor Nr2f2 (COUP-TFII: which was linked to the antiadipogenic hedgehog pathway (acting downstream, by interaction with Gata factors) and could be placed upstream of Cebpa, having an inhibitory effect on its promoter [57]. Both, the Irf and the Nr2f2, studies prove the utility of this (semi) high-throughput approach and highlight its advantage over ChIP-chip studies, which require knowledge of an involved transcription factor and thereby, have reduced potential in the discovery of new transcription factors.

To this end, endeavors are underway to take DNase I hypersensitivity assays to the genome-wide level by combining this technique with hybridization to tiling arrays [98,99] or with massive parallel sequencing [100]. Providing a DNase hypersensitivity map of adipogenesis will be helpful for the adipogenesis community and, in combination with other omics strategies, will broaden our knowledge on the transcriptional landscape during fat cell development.

In a chemical biology approach over 500 compounds from a small-molecule library (BIOMOL) were screened for activator and repressor activities using a 3T3-F442A reporter cell line, that stably expressed luciferase under the control of the adipocyte differentiation-dependent aP2 promoter [101]. Besides known and unknown adipogenesis inhibitors including various retinoids, prostaglandin F and the kinase inhibitor PP1, two positive regulatory compounds, namely harmine and RG14620, were identified by this high-throughput screen. Harmine was selected for further studies and could be determined in this context as cell-type-specific regulator of Pparg expression that mimics the effects of Pparg agonists in-vitro (3T3-F442A, 3T3-L1) and in diabetic mice. Further analyses, however, showed that harmine is not a ligand of Pparg, rather acts *via *a mechanism that involve the inhibition of the Wnt signaling pathway [101]. A structure-function analysis of harmine derivates indicate that the effect on adipogenesis is with one exception limited to harmine. This phenotypic screening of adipocytes is not only a promising approach to the discovery of novel anti-diabetic small molecules with a distinct mechanism of action and side-effect profile, but also to reveal biological processes and factors, which are targeted by these compounds and control the adipogenesis process.

## INFERRING FUNCTION AND MECHANISMS FROM LARGE-SCALE DATA BY COMPUTATIONAL BIOLOGY

Following data generation in omics studies, data preprocessing and normalization is required to extract the data points above the noise level and to submit these data to statistical analyses for the identification of differentially expressed or modulated genes/proteins across samples or experiments. Once the candidate genes are identified, major efforts are directed towards functional validation [102]. The starting point is usually a list of candidates to elicit the biological meaning and the molecular mechanisms mirrored in the activity of the genes. Usually the first step in such analyses is to group genes based on their similarity in expression patterns in several groups. For this purpose a number of different unsupervised clustering methods can be applied (hierarchical clustering, k-means clustering, self organizing maps, principal component analyses) based on appropriate measures indicating the similarity (or distance). Next, in order to obtain the function of the candidate genes Gene Ontology (GO) terms are applied [103]. The GO project provides a controlled vocabulary for describing gene and gene product attributes in any organism in three independent hierarchies: biological process, molecular function, and cellular component. In case of a very common GO term, a high number of genes from the dataset (or cluster) mapping to this GO term, does not necessarily indicate that it is specific to this dataset or cluster. To overcome this drawback, statistical significance is assigned by using Fisher’s exact test or hypergeometric test to proof each GO term within the dataset against the occurrence within all genes (e.g. Refseq database or all elements on the array). This approach could be also applied to other entities like domains, pathways, regulatory sequence motifs (including predicted response elements for transcription factors or target motifs for microRNAs), and gene sets (as within the gene set enrichment analysis (GSEA) [104]). A systematic presentation and analysis of these data in a biological context can be achieved by mapping expression profiles of genes or proteins simultaneously onto major, currently available regulatory, metabolic and cellular pathways (KEGG, Biocarta, GenMAPP) as for example implemented in the PathwayExplorer [105].

In the case of uncharacterized genes, *de novo* functional annotation can be performed on a sequence segment/domain-wise basis. For this purpose several prediction tools need to be integrated and the results can be mapped subsequently onto known pathways, possible cellular roles, and subcellular localizations [22]. Using this approach for DNA microarrays, however, requires finding similar transcript sequences and corresponding protein sequence in selected databases based on the probe sequences (ESTs). Moreover, a major challenge for these computational approaches is not only the construction of (weighted-) gene co-expression networks [106] but also the inference of gene regulatory networks based on sequence, ChIP-chip, and gene expression data and the ultimate integration of the heterogeneous datasets.

## DISCUSSION AND OUTLOOK

Experimental studies using microarrays and proteomics technologies to investigate adipogenesis identified a large and confident set of candidate molecules and putative drug targets in adipocytes. A fraction of these were subsequently characterized in functional studies and not only provided novel mechanistic insights, but also pinpointed target molecules for therapeutic intervention. For example, a recent microarray profiling study identified and validated adipogenic factors including Nr1h3 (LXRalpha) and phospholipid transfer protein (Pltp) as well as candidates for the delicate balance between adipocytes and osteoblasts in bone marrow [35]. One of the candidates, oxytocin receptor (Otr), was subsequently verified in preclinical and clinical studies showing that oxytocin administration holds promise as a potential therapy for osteoporosis [107].

Omics technologies enabled for the first time a comprehensive assessment of the various molecular species in a cell and sparked a number of studies. The choice of a specific technology to address certain biological questions has to be weighted depending on several technological, scientific, and economic parameters. Currently, microarrays are widely used due to the maturity of the technology, robustness of the instruments, the relative inexpensiveness, the inherent sensitivity, and the availability of bioinformatics solutions to manage and analyze the data. In other studies, measurement of RNA levels might not be sufficient and proteomics experiments may be required. In contrast to microarrays, the proteomics technologies (MALDI-TOF or LC-MS/MS) are not as sensitive, less complete (only a fraction of the proteome is detectable), and generate a wealth of data, which is difficult to manage and analyze. Hence, currently only a handful of labs are able to apply high-throughput proteomics technologies and deal with the data.

Large-scale experiments are prone to low or absent quantitative measurements of molecules. For the identification of enriched or differentially regulated biological processes a moderate number of spurious detections is tolerable. For example based on the gene expression profiles from several microarray studies, candidate genes shown in Fig. (**2**) that are relevant for adipogenesis were not detected by any (e.g. Klf15) or only by some (e.g. Pnpla2) of the presented studies. This demonstrates that integration of several datasets and meta-analysis is instrumental. Another issue is the consistency or inconsistency of gene expression data. The confidence of the selected candidates for further functional studies is increasing if there are consistent results over several studies. This applies even more to a situation, where the analysis is based on studies from different platforms, technologies or omics-data. For instance haptoglobin (Hp) was differentially expressed in adipocytes versus preadipocytes [15,16,18,85], was identified as Pparg target [84], as well as Cebpa target [85], and was identified as secreted protein by a proteomics study [72]. It should be noted that the generation of large-scale data is connected with high costs, different degrees of complexity and experimental issues (e.g. instrumental effort for proteomics studies or validation of protocols and antibodies for detection of protein-DNA binding studies). The major advantage comes with the high number of detected molecules and the possibility to perform combined analyses.

Integrating data from various different omics technologies enables us to draw a broader picture of a cells behavior and of the implication of certain (experimental) treatments or environmental signals. This will ultimately lead to systems biology, an emerging interdisciplinary study field that focuses on the complex interactions in biological systems [108]. One goal of systems biology is to understand how genome-encoded parts interact to produce quantitative phenotypes. Systems biology has the power to transform the way biology and medicinal chemistry has been viewed classically by way of dealing with biological entities on the systemic level rather than focusing on a system which is simplistically reduced to a small number of parts. Although so far major discoveries were made mostly with microbial systems, this approach will be of substantial interest also for mammalian systems. However, the integration of diverse omics data sets poses major challenges to researchers (in particular bioinformaticians) and computational infrastructures [109]. Further, it demands standards that make data sets from different sources (labs, platforms, technologies) reliable, comparable, and, ultimately, amenable for integration on a broad scale [110]. In this context it will be inevitable to apply mathematical modeling in order to interpret the flood of data. Mathematical modeling is an important addition to the toolbox of molecular techniques and it will be important to train biologists and medicinal chemists so they can use these methods much like any other wet lab method.

All afore mentioned fields are under constant development, inevitably spawning major breakthroughs that make systems biology and its applications more and more palpable. As an example, next-generation sequencing technologies - also known as high-throughput sequencing, deep sequencing or third generation sequencing (available platforms: Solexa (Illumina), 454 (Roche) and SOLiD (ABI)) - are about to shift omics strategies that rely on hybridization on microarrays to sequencing of the molecule under question [111,112]. In the case of transcriptomics several recent publications proved the utility of the RNA-seq technology to assess (m)RNA levels in different applications, highlighting its advantages over array-based methods, namely: higher signal-to-background-ratio, lower detection limit, unbiased measurements, unambiguous assignment of measured sequences, and quantitative linearity over a broader range [111,113-115]. For genome-wide location analysis next-generation sequencing was employed successfully to sequence DNA material from chromatin immunoprecipitation experiments having the advantage of an unbiased detection of transcription factor binding in the whole genome, not only in promoter regions spotted on promoter arrays [86,87,116-118]. If throughput and quality increase and prices per sequencing read decrease as predicted (the “$1000 genome”) these sequencing technologies might become more wide-spread and, owing to their technical advantages over hybridization-based approaches, might become the gold standard in measuring DNA and RNA specimen on a genome-wide scale.

In summary, omics technologies generated plethora of data and provided novel mechanistic insights into adipogenesis which can be ultimately exploited for developing novel drugs for the treatment of obesity. It became also evident that we are only at the beginning of drawing the complete picture of the complex cellular process of fat cell commitment and differentiation, and that further integrative omics-approaches will be necessary to elucidate the molecular network controlling the cell fate.

## Figures and Tables

**Fig. (2):**
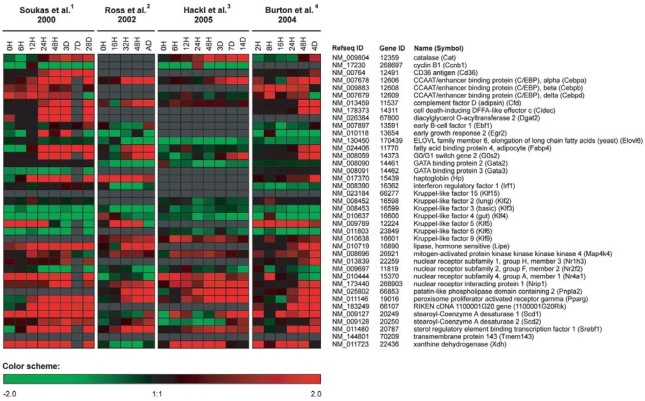
Gene expression profiles of selected candidates from 3T3-L1 time series experiments. ^1^Raw data were downloaded from Gene Expression Omnibus (GEO) GSE6794, normalized using GCRMA and annotated with recent annotation files for Mu11kA and Mu11kB downloaded from the Affymetrix website. Log2ratios are visualized for each time point in relation to the preconfluent stage; ^2^Normalized expression data from Ross *et al*. 2002 were downloaded from the authors’ website  http://wwwpersonal. umich.edu/~macdouga/MacDougaldLab.html  and data set were filtered for genes, which did not show an average difference of >100 in at least one condition. Relative expression levels (log2ratios) were related to the 3T3-L1 preconfluent stage and averaged over two measurements; ^3^Data for Hackl *et al*. 2005 was derived from ArrayExpress (E-MARS-02), normalized as stated by the authors and ESTs were annotated using a current Refseq version. Relative expression levels (log2ratios) from different time points were related to the preconfluent state and averaged over 3 independent experiments; ^4^Raw data (CEL files) were downloaded from  http://wwww.arcs.uams.edu/ directory/microarray.asp , normalized using GCRMA and annotated using an annotation file provided by Affymetrix for U74Av2 mouse microarrays. Relative gene expressions were related to time point 0 immediately before hormonal induction and data were averaged over 2 replicated experiments; data were color coded according to the color scheme added to the figure).

**Table 1:** Large-Scale Expression Profiling Studies During Adipogenesis

Study	Cell type	Treatment	Time points	Array
**Expression profiling studies on 3T3-L1 cell line**				
Guo *et al*. 2000 [14]	3T3-L1	DMI	0h, 6d	18k mouse cDNA filter arrays
Soukas *et al*. 2000 [15]	3T3-L1	DMI	PC,0h,6h,12h,24h,2d,3d, 4d, 7d,28d	Affymetrix mouse Mu11k
Ross *et al*. 2002 [16]	3T3-L1	DMI|DMI+Wnt-1↑	0h, 16h, 32h, 2d, d14	Affymetrix mouse U74A
Burton *et al*. 2002 [17]	3T3-L1	DMI	0h,2h,8h,16h,24h	AffymetrixmouseMu11k
Burton *et al*. 2004 [18]	3T3-L1	DMI|DMI+TSA	0h,2h,8h,16h,24h,d2,d4	Affymetrix mouse U74A
Jessen at al. 2002 [19]	3T3-L1	DMI	0h, 1d, 4d, 7d	Affymetrix Mu11kB| U74Av2
Gerhold *et al*. 2002 [20]	3T3-L1	DMI+TZD|nTZD	0h, PA 6hT, PA 48hT, AD 6hT, AD 48hT	4k Affymetrix mouse mu6800
Hsiao *et al*. 2004 [21]	3T3-L1	TZDs	AD 24hT	Affymetrix mouse U74Av2
Hackl *et al*. 2005 [22]	3T3-L1	DMI	PC,0h,6h,12h,24h,2d,3d,4d,7d,14d	27K mouse cDNA microarray
Liu *et al*. 2005 [23]	3T3-L1	DMI|DI		Affymetrix mouse MOE430A
Pantoja *et al*. 2005 [24]	3T3-L1	D↔M I| M↔D I		38 k MEEBO microarray oligo set
**Adipogenesis expression studies on other mouse cell models including over-expression and knock out strategies**				
Huo *et al*. 2006 [25]	3T3-F442A	GH		Affymetrix mouse U74Av2
Akerblad *et al*. 2005 [26]	NIH-3T3	Ebf1↑|Pparg↑		Affymetrix mouse U74Av2
Baudry *et al*. 2006 [27]	PKB-/-MEF			22k Affy mouse MOE430A
Christian *et al*. 2005 [28]	RIPKO			Affymetrix mouse MOE430 2.0
Otto TC *et al*. 2007 [29]	C3H10T1/2	Bmp4↑		Affymetrix mouse MOE430 2.0
Tseng *et al*. 2005 [30]	IRSKO PA			Affymetrix mouse U74Av2
Timmons *et al*. 2007 [31]	wPA, bPA		0d, 1d, 4d, 8d	Affymetrix mouse U74Av2
**Adipogenesis of human cell models**				
Hung *et al*. 2004 [32]	hMSC	DMI	0h,3d	8k human Universochip
Sekiya *et al*. 2004 [33]	hMSC		0h,1d,7d,14d,21d	12k Affymetrix human U95Av2
Nakamura *et al*. 2003 [34]	hMSC			3.4k focused cDNA microarray
Scheideler *et al*. 2008 [35]	hMADS	DMI+ROS	PC,-2d, 0h, 8h, 1d, 2d, 5d, 10d, 15d	30k human spotted oligo array
Urs *et al*. 2004 [36]	PA|AD			19K human cDNA microarray

Abbreviations: Preadipocyte (PA), White and brown preadipocyte (wPA and bPA), Adipocyte (AD), Preconfluent (PC), Dexamethasone+Dethylisobutylxanthine+Insulin (DMI), Trichostatin A (TSA), Rosiglitazone (ROS), Thiazolidione (TZD), none TZD compounds (nTZD), Human mesenchymal stem cells (hMSC), Human multipotent adipose-derived stem cells (hMADS), Insulin receptor substrate knock-out (IRSKO).

**Table 2:** High-Throughput and Medium-Throughput Methods to Identify Adipogenic Candidate Genes

Study	Cell type	Treatment	Metdod used	Identified candidates
Adachi *et al*. 2007 [71]	3T3-L1	DMI	LC-MS/MS	3,287 proteins
Eguchi *et al*. 2008 [97]	3T3-L1	DMI	DNase I Hypersensitivity assay	Interferon Regulatory Factors 1-9, Nr2f2 (COUP-TFII)
Puri *et al*. 2008 [90]	3T3-L1	DMI	RNA interference screen	Nrip1, Scd2, Cidec, Map4k4, among others
Waki *et al*. 2007 [101]	3T3-F442A	>500 compounds	Screening of small molecule library	Harmine and RG14620
Nakachi *et al*. 2008 [84]	3T3-L1	DMI	ChIP-chip	Tmem143, Hp, 1100001G20Rik
Lefterova *et al*. 2008 [85]	3T3-L1	DMI	ChIP-chip (whole-genome tiling)	Listed in supplemental material of publication
Nielsen *et al*. 2008 [86]	3T3-L1	DMI	ChIP-seq	Listed in supplemental material of publication
Hamza *et al*. 2009 [87]	3T3-L1	DMI+R	ChIP-PET	Plin, Pcx, Mgst1, Gpd1, Pim3, Mnk2, Agt, Fsp27, Adipogenin, Pdzrn3
Wakabayashi *et al*. 2009 [88]	3T3-L1	DMI	ChIP-chip and ChIP-seq	SET domain proteins (Setd8, Setdb1, Setd5)

Abbreviations: Dexamethasone+Dethylisobutylxanthine+Insulin (DMI), Rosiglitazone (R), Liquid chromatography- tandem mass spectrometry (LC-MS/MS), Chromatin immuno-precipitation on chip (ChIP-chip), ChIP sequencing (ChIP-seq), ChIP pair end-tagging (ChIP-PET).

## ABBREVIATIONS

WAT: = White adipose tissue
MEFs: = Mouse embryo fibroblasts
hMADS: = Human multipotent adipose-derived stem cells
Pparg: = Peroxisome proliferators activated receptor gamma
Cebpa: = CCAAT/enhancer-binding protein alpha
Klf: = Krüppel like factor
Scd: = Stearoyl-CoA desaturase
ChIP: = Chromatin immunoprecipitation
MALDI-TOF/MS: = Matrix-assisted laser desorption/ionization – time of flight/mass spectrometry
LC-MS/MS: = Liquid chromatography - Tandem mass spectrometry
DMI: = Dexamethasone, isobutylmethylxanthine, and insulin
qPCR: = Quantitative real-time reverse transcriptase polymerase chain reaction
GO: = Gene Ontology
GSEA: = Gene set enrichment analysis
